# International Evidence on the COVID-19 Deaths of People Who Live in Long-Term Care Facilities

**DOI:** 10.1093/geroni/igab046.1577

**Published:** 2021-12-17

**Authors:** Joseba Zalakain, Elizabeth Lemmon, David Henderson, Amy Hsu, Andrea Scmidt, Greg Arling, Florien Kruse, Adelina Comas-Herrera

**Affiliations:** 1 SIIS Centro De Documentación Y Estudios, Donostia San Sebastián, Pais Vasco, Spain; 2 University of Edinburgh, Edinburgh, Scotland, United Kingdom; 3 University of Edinburgh, NINE Edinburgh BioQuarter, Scotland, United Kingdom; 4 Bruyère Research Institute, Ottawa, Ontario, Canada; 5 Austrian National Public Health Institute (Gesundheit Österreich GmbH, GÖG), Vienna, Wien, Austria; 6 Purdue University, West Lafayette, Indiana, United States; 7 Radboud University Medical Center, Nijmegen, Gelderland, Netherlands; 8 London School of Economics and Political Science, London School of Economics and Political Science, England, United Kingdom

## Abstract

The COVID-19 pandemic has had a disproportionate impact, in terms of mortality, on people who live in Long-Term Care Facilities (LTCFs). This study involved compiling data on number of deaths of people who live in LTCFs and analyzing the extent to which differences between countries could be attributed to measures taken to control the spread of COVID-19 to LTCFs or to other factors. The study found that differences in how the data is collected make international comparisons difficult but that there is a clear correlation between number of COVID-19 deaths of residents in LTCFs and number of COVID-19 deaths of people living in the community. The study also found that countries that experienced a particularly high number of deaths in LTCFs during the first COVID-19 wave tended to have lower relative mortality in LTCFs in the subsequent waves, which potentially could be attributed to learning from the initial shock.

